# Treatment of Pulmonary Arterial Hypertension in Lithuania: Current Situation and Analysis of Survival of Patients Treated with Different Treatment Regimens

**DOI:** 10.3390/jcm15072795

**Published:** 2026-04-07

**Authors:** Skaidrius Miliauskas, Deimante Hoppenot, Ieva Dimiene, Egle Grigoniene, Lina Gumbiene, Irena Nedzelskiene, Mangirdas Vaizgela, Egle Ereminiene

**Affiliations:** 1 Department of Pulmonology, Lithuanian University of Health Sciences, LT-44307 Kaunas, Lithuania; deimante.hoppenot@lsmu.lt (D.H.); ieva.dimiene@lsmu.lt (I.D.); 2 Pulmonary Hypertension Center of Hospital of Lithuanian University of Health Sciences Kauno Klinikos, LT-50161 Kaunas, Lithuania; egle.ereminiene@lsmu.lt; 3 European Reference Network for Rare Respiratory Diseases (ERN-LUNG), DE-60596 Frankfurt am Main, Germany; egle.grigoniene@santa.lt (E.G.);; 4 Pulmonary Hypertension Competence Center, Vilnius University Hospital Santaros Klinikos, LT-08406 Vilnius, Lithuania; 5 Clinic of Cardiac and Vascular Diseases, Institute of Clinical Medicine, Faculty of Medicine, Vilnius University, LT-03101 Vilnius, Lithuania; 6 Department of Dental and Oral Diseases, Lithuanian University of Health Sciences, LT-44307 Kaunas, Lithuania; irena.nedzelskiene@lsmu.lt; 7 Economics and Data Analytics Programme, International School of Management, LT-01103 Vilnius, Lithuania; mangirdas@hta-solutions.com; 8 Institute of Cardiology, Lithuania University of Health Sciences, LT-50103 Kaunas, Lithuania; 9 Heart Centre, Medical Academy, Lithuanian University of Health Sciences, LT-50140 Kaunas, Lithuania

**Keywords:** PAH, treatment, PAH-specific medications, Lithuania, survival

## Abstract

**Background/Objectives**: Since 2015, pulmonary arterial hypertension (PAH)-specific medications have been fully reimbursed in Lithuania. To describe the current situation of PAH treatment in the country and to determine survival during different PAH treatment regimens. **Methods**: The data from the Institute of Hygiene and the State Data Agency of Lithuania cases with administrative codes I27.0 and I27.8 have been evaluated. **Results**: In 2025, 225 confirmed cases of PAH were treated with PAH-specific medications in two PH centers. At least one PAH-specific medication was prescribed to 163 (72.4%) female and 62 (27.6%) male patients. Among these, 96 (42.7%) received sildenafil monotherapy, 82 (36.4%) received a combination of sildenafil and an ERA, 36 (16.0%) were on triple PAH-specific therapy (including selexipag or treprostinil), and 11 (4.9%) received other regimens due to specific medical considerations. The age of adults treated with sildenafil monotherapy vs. other therapies was 63.9 ± 14.8 (*n* = 117) and 51.5 ± 17.3 (*n* = 116) years, respectively (*p* < 0.05). A total of 191 PAH patients who received targeted therapy died during the observational period 2017–2025. Of these, 105 received monotherapy, 57 sildenafil and endothelin receptor antagonist and 29 triple therapies (treprostinil [*n* = 19], selexipag [*n* = 6], or inhaled iloprost [*n* = 4] were prescribed as the third drug). Patients who died and received triple therapy were younger than those on mono- and dual therapy (age at diagnosis 45.0 ± 21.6, 67.2 ± 14.7 and 61.6 ± 16.3 years, respectively, *p* < 0.01). Survival was longer in patients on dual therapy compared with monotherapy (43.1 ± 28.1 vs. 31.7 ± 25.0 months, *p* = 0.04), and the longest was in those receiving triple therapy (59.9 ± 29.4 months; *p* < 0.05). **Conclusions**: The availability of reimbursed medications dramatically increased the number of treated PAH cases in Lithuania. In 2025, most of the PAH patients received sildenafil monotherapy. Patients treated with sildenafil only were significantly older than the rest of cohort. In the survival analysis, combination PAH therapies were more often prescribed to younger patients and were associated with longer duration of life than monotherapy.

## 1. Introduction

Pulmonary hypertension (PH) is a condition marked by increased pressure in the pulmonary circulation that elevates the workload of the right heart and can lead to heart failure. PH is very heterogeneous, may complicate the majority of respiratory, cardiovascular or systemic diseases and is not rare. PH can be found in about 1% of the world’s population and increases up to 10% in those aged over 65 [1]. Pulmonary arterial hypertension (PAH) is a chronic and progressive disease with the narrowing of small pulmonary arteries, increased pulmonary vascular resistance (PVR), causing right heart failure, and subsequently death [2]. In economically developed countries, registry data show that PAH incidence and prevalence are 5.8 and 47.6–54.7 cases per one million adults [3,4]. PAH patients are hemodynamically characterized by precapillary PH with no known other causes of precapillary PH, such as chronic thromboembolic PH (CTEPH), PH associated with lung diseases, or unclear and/or multifactorial mechanisms [5,6,7]. PH patients usually seek doctor’s attention due to non-specific symptoms such as dyspnea, fatigue, chest pain, and syncope, which consequently delays the establishment of a proper diagnosis and the initiation of treatment [8,9].

The 2022 European Society of Cardiology and European Respiratory Society guidelines recommend guiding treatment decisions for PH by underlying etiology. Targeted therapies are recommended for PAH and CTEPH patients, and not for those with PH associated with left-heart or respiratory diseases. Once PAH is diagnosed, the recommended treatment strategy should be based on calculated risk of death and the presence of additional cardiopulmonary comorbidities [7]. The current treatment strategy for PAH underlines the importance of early initiation of the combined PAH-targeted therapies to reduce mortality and achieve low-risk status. This status is defined when a 1-year mortality rate is less than 5% [7,10,11]. Until recently there were three main classes of PAH-specific medications: (1) endothelin receptor antagonists (ERAs); (2) drugs that enhance the nitric oxide pathway—phosphodiesterase type 5 inhibitors (PDE5i) and soluble guanylate cyclase (sGC) stimulators; (3) drugs that work through the prostaglandin I_2_ (PGI_2_) pathway, including prostacyclin itself, analogs of PGI_2_, and agonists of the PGI_2_ receptor [12,13]. Recently, sotatercept, a novel activin signaling inhibitor, has been approved for clinical use. However, the availability of this drug is still limited in most Central and Eastern European countries [3,14,15,16,17].

PAH-specific medications have significantly improved patients’ survival. Before targeted treatments became available, survival with PAH was very poor. The U.S. registry study from 1991 reported that individuals diagnosed with idiopathic PAH (previously called primary pulmonary hypertension) had a median survival of about 2.8 years [18]. More recent data show that median survival now surpasses five years [19,20,21]. Although the use of combination treatment increased between 2010 and 2019, the majority of patients with PAH were still treated with a single medication during this time [22].

In most Central and Eastern European countries, therapies specifically targeting PAH were not formally included in reimbursed treatment regimens until the early twenty-first century, and the availability of advanced diagnostic tools and targeted treatments was significantly delayed compared with Western Europe. Nowadays, the availability of oral PAH-specific treatment in these countries is improved. Additionally, patients have access to parenteral prostanoids [3]. Table 1 summarizes epidemiological findings and available treatment options in various Central and Eastern European countries, drawing on data from observational studies, registries, and expert consensus.

In Lithuania, PAH-specific medications have been fully reimbursed since 2015 only, which has significantly improved treatment accessibility. The most recently introduced medications were macitentan and selexipag (both since 2019). Activin signaling inhibitor sotatercept was temporarily available in 2024 in the early access program. Now, access is limited and granted only through individual requests to the Commission for Very Rare Diseases. According to reimbursement rules, initial PAH treatment should begin with sildenafil monotherapy. If the effect is insufficient, other medications may be added at the discretion of the treating physician, following discussion with the multidisciplinary PH team and risk assessment very soon after. Only patients classified as World Health Organization (WHO) functional class III are eligible to initiate and later continue treatment. In Lithuania, there are two PH centers, both located in university hospitals: in the Hospital of the Lithuanian University of Health Sciences Kauno Klinikos and in Vilnius University Hospital Santaros Klinikos. Reimbursed PAH-specific medications can only be prescribed in these centers, where diagnosis of PAH is confirmed according to the international guidelines. In this study, we aimed to provide an overview and describe the current situation of PAH treatment in Lithuania and to determine survival during different PAH treatment regimens. The data of PAH treatment in Lithuania have not previously been published in any form. Also, there is a shortage of real world data concerning the survival of patients treated with PAH-specific medications.

## 2. Materials and Methods

This study is observational and descriptive. The data on medication prescriptions, age during PAH diagnosis, gender and survival on PAH treatment from the Institute of Hygiene and the State Data Agency of Lithuania for patients with PAH administrative codes I27.0 and I27.8, based on the International Statistical Classification of Diseases and Related Health Problems, 10th Revision, Australian Modification (ICD-10-AM), have been analyzed. For statistical analysis, we used IBM SPSS Statistics for Windows, version 30.0.0.0 (IBM Corp., Armonk, NY, USA). These codes include idiopathic PAH, connective tissue disease-associated PAH, portopulmonary hypertension, and congenital heart disease-associated PAH. To analyze differences in treatment groups, Student’s *t*-test for two independent samples was used. The Smirnov–Kolmogorov test was used for assumptions of normality. In the survival analysis, treatment groups were compared using two-sided Welch’s *t*-tests to account for unequal sample sizes and variances. Bonferroni correction was applied for three pairwise comparisons. Continuous variables are presented as mean and standard deviation. Kaplan–Meier survival analysis was performed to evaluate survival differences between treatment groups. A Cox proportional hazards regression model was used to assess the association between treatment strategy and survival while adjusting for age. Patients were considered treated if PAH medications were prescribed within 3.1 months before death.

## 3. Results

**Figure 1:**
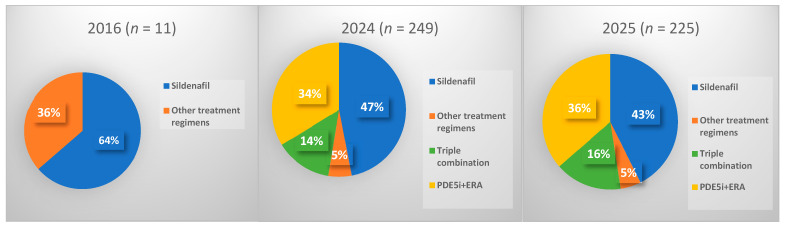
Distribution of treatment regimens in PAH cases (ICD-10-AM I27.0 or I27.8) in 2016, 2024 and 2025. Abbreviations: PAH—pulmonary arterial hypertension, ICD-10-AM—International Statistical Classification of Diseases and Related Health Problems, 10th Revision, Australian Modification, PDE5i—phosphodiesterase type 5 inhibitor, ERA—endothelin receptor antagonist, *n*—number of patients.

In 2016 only 11 patients diagnosed with PAH (I27.0 or I27.8 codes) were treated with reimbursed PAH-specific medications. In 2024, the number increased to 249, corresponding to 86.3 cases per 1,000,000 inhabitants. At least one PAH-specific medication was prescribed to 178 (71.5%) female and 71 (28.5%) male patients. Among these, 117 (47.0%) received sildenafil monotherapy, 84 (33.7%) received a combination of sildenafil and an ERA, 34 (13.7%) were on triple PAH-specific therapy (including selexipag or treprostinil), and 14 (5.6%) received other regimens due to specific medical considerations. In 2025, 225 patients with PAH were treated. At least one PAH-specific medication was prescribed to 163 (72.4%) female and 62 (27.6%) male patients. Among these, 96 (42.7%) received sildenafil monotherapy, 82 (36.4%) received a combination of sildenafil and an ERA, 36 (16.0%) were on triple PAH-specific therapy (including selexipag or treprostinil), and 11 (4.9%) received other regimens due to specific medical considerations. In Figure 1, the distribution of treatment regimens in PAH cases in 2024 and 2025 is compared with that in 2016.

**Figure 2:**
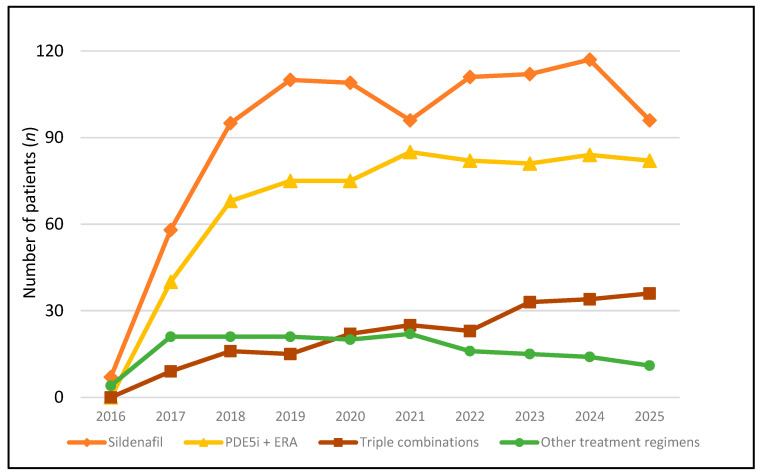
Number of patients with ICD-10-AM codes I27.0 and I27.8, who were prescribed PAH-specific monotherapy or combination therapies between 2016 and 2025. Abbreviations: ICD-10-AM—International Statistical Classification of Diseases and Related Health Problems, 10th Revision, Australian Modification, PAH—pulmonary arterial hypertension, PDE5i—phosphodiesterase type 5 inhibitor, ERA—endothelin receptor antagonist.

After the sharp increase in the number of patients treated with PAH-specific medications at the beginning of reimbursement, the number of treated patients has remained almost stable in Lithuania since 2018 (Figure 2).

In 2024, younger adults with PAH were more likely to receive combination therapy. The mean age of all patients receiving sildenafil monotherapy was 63.9 ± 14.8 years, and the mean age of patients receiving other PAH therapies was 52.2 ± 16.7 (*p* < 0.05) (Table 2). In 2025 the situation did not change. The mean age of all patients receiving sildenafil monotherapy was 65.1 ± 15.1 years, while that of those receiving other PAH therapies was 51.7 ± 16.4 (*p* < 0.05) (Table 3).

A total of 191 PAH patients who received targeted therapy died during the observational period 2017–2025. Of these, 105 received monotherapy, 57 sildenafil and endothelin receptor antagonist and 29 triple therapy (treprostinil [*n* = 19], selexipag [*n* = 6], or inhaled iloprost [*n* = 4] were prescribed as the third drug). Patients receiving triple therapy were younger than those on mono- and dual therapy (age at diagnosis 45.0 ± 21.6, 67.2 ± 14.7 and 61.6 ± 16.3 years, respectively, *p* < 0.01), with no difference in age between mono- or dual therapy. Survival was longer in patients on dual therapy compared with monotherapy (43.1 ± 28.1 vs. 31.7 ± 25.0 months, *p* = 0.04), and longest in those receiving triple therapy (59.9 ± 29.4 months; *p* < 0.05) (Table 4 and Table 5).

**Figure 3:**
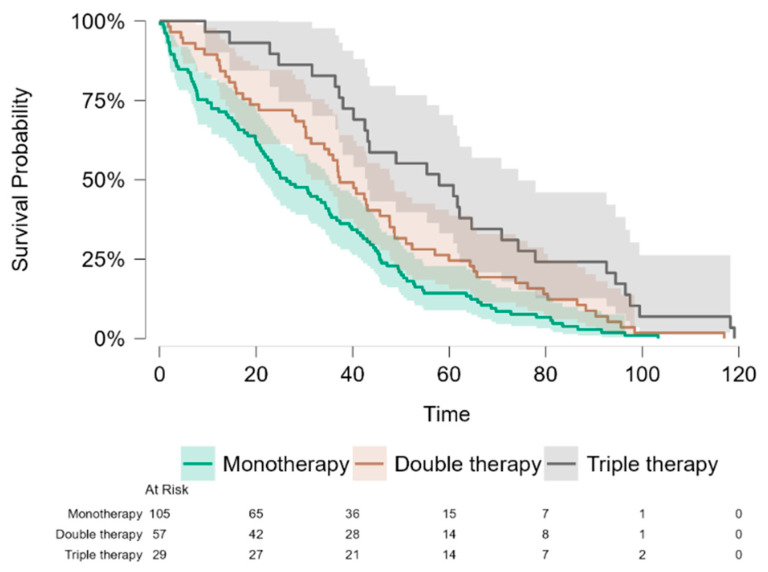
Kaplan–Meier survival curves stratified by treatment strategy.

Kaplan–Meier survival analysis was performed to evaluate survival differences between treatment groups. Survival curves demonstrated significant differences between treatment strategies (log-rank test, *p* < 0.001), with the longest survival observed in the triple therapy group and followed by double therapy and monotherapy (Figure 3).

Cox proportional hazards regression models were used to assess the pairwise associations between treatment strategies and survival risks while adjusting for age. Compared with double therapy, monotherapy was associated with a significantly increased risk of death (*p* = 0.014), while triple therapy was associated with a significantly reduced risk of death (*p* = 0.015). Compared with triple therapy, monotherapy was associated with a significantly increased risk of death (*p* < 0.001). Age was not significantly associated with survival (HR = 0.97, 95% CI 0.99–1.01, *p* = 0.433). To evaluate the potential confounding effect of age, we compared models with and without adjustment for age. The hazard ratios for treatment groups remained stable after adjustment (monotherapy vs. double HR 1.47 vs. 1.54; triple vs. double HR 0.58 vs. 0.55; monotherapy vs. triple HR 2.54 vs. 2.79), indicating that age did not confound the association between treatment and survival. Detailed comparisons of hazard ratios with and without age adjustment are presented in Table 6.

## 4. Discussion

This is the first publication with data on PAH treatment and survival analysis of patients diagnosed with PAH in Lithuania. Although international guidelines recommend initial combination therapy for most patients with PAH, monotherapy remains the predominant treatment approach in Lithuania, thereby reflecting a gap between guideline recommendations and real-world clinical practice.

In an analysis of COMPERA, a large European pulmonary hypertension registry comprising 2531 patients, Hoeper et al. [22] evaluated the use of combination therapy and survival outcomes in newly diagnosed PAH cases from 2010 to 2019. Early combination therapy—initiated within three months of diagnosis—increased from 10% in 2010 to 25% in 2019. One year after diagnosis, the proportion of patients receiving combination therapy rose from 27.7% to 46.3%. Despite this upward trend, overall adoption of combination therapy remained relatively low. Notably, approximately two-thirds of patients under 65 years old received combination therapy within a year of diagnosis, compared with only about one-third of older patients. The authors suggest that this may reflect clinical inertia among physicians, as well as a scarcity of clinical trials involving older patients with comorbid conditions. However, they emphasize that clinicians should not be hesitant to initiate or implement early oral combination therapy in patients with PAH [22]. With regard to clinical evidence, several large registry studies and randomized controlled trials did not demonstrate a direct survival advantage of initial combination therapy over monotherapy [20,22,23,24]. Nevertheless, current guidelines are grounded in prospective randomized controlled trials that demonstrate significant benefits across multiple clinically relevant outcomes [7,25,26].

In the new Compera analysis of different treatment strategies and survival of patients with connective tissue diseases and PAH [27], survival was significantly worse in systemic sclerosis patients. The authors underline that long-term survival can be improved by prescribing initial combination therapy with sildenafil and endothelin receptor antagonist for this group of patients compared with monotherapy.

In the ongoing, multicenter, prospective, observational EXPOSURE study [28] most patients diagnosed for ≤6 months during enrollment initiated treatment as monotherapy (48%) or double therapy (43%). The patients who were diagnosed more than 6 months prior were included in the study on initiation of combination therapy and most of them did not change treatment regimen later. The authors suggest a shift towards PAH combination therapy and the changing of real-world treatment according to the current guideline recommendations. 

In Lithuania, the option to prescribe a combination therapy immediately after PAH diagnosis is still restricted by reimbursement policies. Patients must initially be treated with sildenafil, and only sequential therapy is allowed, which can be started very early. Those patients are managed in the PH centers only by pulmonologists and cardiologists, where PAH diagnosis is confirmed with right-heart catheterization and additional investigations. In our study, we observed that sildenafil monotherapy has remained the predominant treatment choice over the years and was more likely to be prescribed for elderly patients. Furthermore, we assume that the frequent use of sildenafil monotherapy (47% and 43%) observed in 2024 and 2025, particularly among older patients, is potentially explained by the fact that a substantial proportion of these patients had concomitant cardiovascular or chronic lung diseases and physicians tried to prescribe “less medications”.

Most previously published studies focus on survival rates. Evaluations of changes in age at death among patients with PAH are relatively uncommon. We identified a couple of studies in which this was assessed. An earlier Nordic study demonstrated that the age at death among patients with Eisenmenger syndrome rose from 27.7 years during 1977–1992 to 46.3 years during 2006–2012 [29]. However, other subtypes of group I PAH were not represented in the study. Meanwhile, a systematic analysis published in 2025 revealed that the burden of PAH has progressively improved, as evidenced by a 38.2% decrease in age-standardized years of life lost (YLLs) in 2021 compared with 1990. YLLs in this analysis were calculated directly from the age at death [30].

In studies from France and the UK, the management of patients with PAH at the last follow-up before death was analyzed [31]. The French registry and the UK cohort data were similar. Only 9% of patients (160 out of 1924 and 50 out of 558 respectively) were receiving triple therapy including subcutaneous or intravenous prostaglandin therapy, and 43 and 44% received dual oral therapy at the time of death. In our cohort, 19 patients or 9.9% (out of 191) received treatment with parenteral treprostinil and 57 or 29.8% received sildenafil and endothelin receptor antagonist.

The duration of life in our cohort during different PAH treatments was influenced by the treatment chosen by the managing physician. As we can see, survival was longer in patients on dual therapy compared with monotherapy, and longest in those receiving triple therapy. According to our data, the patient’s age was not significantly associated with survival. Currently we do not have survival data according to the different disorders associated with PAH.

Finally, we analyzed the cohort of patients in Lithuania with administrative ICD-10-AM codes I27.0 and I27.8, and it is important to note that these codes may also encompass other forms of PH, such as combined pre- and post-capillary PH (CpcPH) or some cases of CTEPH. We admit that the changes in duration of life over time may also have been improved not only by availability of PAH-specific therapies, but also demographic shifts, improved general healthcare, coding practices, and even the inclusion of older patients.

## 5. Limitations of the Study

There are some limitations to our study, which is observational and descriptive. Firstly, although PAH diagnosis was confirmed in the PH centers, inclusion in the study was based on the administrative ICD-10-AM codes I27.0 and I27.8 in the Institute of Hygiene and the State Data Agency of Lithuania. Some patients with CTEPH or CpcPH might have been encoded with ICD-10-AM code I27.8. We cannot rule out the potential of missing or wrongly entered data, which may have influenced the results. Furthermore, based on data from the Institute of Hygiene and the State Data Agency of Lithuania, in 2016 a total of 11 patients in Lithuania were reported as receiving specific PAH pharmacotherapy; however, unofficial estimates from two Lithuanian dedicated PH centers suggest that the actual number was higher, which may reflect delays in data reporting or other systematic factors. We need a national registry and patient-level clinical data as the primary next step. The risk of death cannot also be assessed from the data provided by the State Data Agency.

Another important limitation is related to statistical analysis. Age was included in the Cox proportional hazards model as a continuous variable to adjust for its potential confounding effect on the association between treatment strategies and survival. Although age was not statistically significant in the multivariable analysis, this does not exclude the possibility of residual confounding. Other factors influencing survival, such as comorbid conditions, could not be accounted for due to limited data availability.

We also admit that there is limited external validity of the results and clarify that findings may not be applicable beyond Lithuania or similar healthcare systems.

## 6. Conclusions

The availability of reimbursement of PAH medications dramatically increased the number of reimbursed prescriptions. In 2024 and 2025, most PAH patients were treated with sildenafil monotherapy. PAH patients treated with sildenafil only were significantly older than the rest of cohort. In the survival analysis, combination PAH therapies were more often prescribed to younger patients and were associated with longer duration of life than monotherapy.

## Figures and Tables

**Table 1:** Epidemiological data of Pulmonary Arterial Hypertension and Availability of Targeted Treatments in Central and Eastern Europe (reproduced with permission from Kopeć G et al. [3]).

	Croatia	Czech Republic	Latvia	Lithuania	Poland	Romania	Slovakia	Slovenia
**Number of patients with PAH/million adults**	41.2 ^a^	49.6 ^a^	45.7 ^a^	55.8 ^a^	30.8 ^a^	50 ^b^	43 ^b^	35 ^b^
**New diagnosis of PAH per year/million adults**	5–6 ^b^	8.5 ^a^	9.0–12.04 ^a^	7–8 ^b^	5.2 ^a^	4–5 ^b^	5.4 ^b^	5 ^b^
**National PAH registry**	No	Yes	Yes	Yes	Yes	No	No	No
**Reimbursed therapies**								
**Bosentan po**	Yes	Yes	Yes	Yes	Yes	Yes	Yes	Yes
**Macitentan po**	Yes	Yes	No	Yes	Yes	Yes	Yes	Yes
**Ambrisentan po**	No	Yes	Yes	Yes	No	No	Yes	Yes
**Sildenafil po**	Yes	Yes	Yes	Yes	Yes	Yes	Yes	Yes
**Tadalafil po**	Yes	Yes	Yes	No	No	No	Yes	No
**Riociguat po**	Yes	Yes	No	Yes	Yes	Yes	Yes	Yes
**Treprostinil sc/iv**	Yes	Yes	Yes	Yes	Yes	Yes	Yes	Yes
**Treprostinil inh**	No	No	No	No	No	No	No	No
**Treprostinil po**	No	No	No	No	No	No	No	No
**Epoprostenol iv**	No	Yes	No	No	Yes	No	Yes	Yes
**Selexipag**	Yes	Yes	Yes	Yes	Yes	Yes	Yes	Yes
**Iloprost iv**	No	No	No	No	No	No	No	Yes
**Iloprost inh**	Yes	Yes	Yes	Yes	Yes	No	Yes	Yes
**Sotatercept sc**	Yes	Yes ^c^	No	No	No	No	No	Yes ^c^
**Double oral combination:** **ERA + PDE5i**	Yes	Yes	Yes	Yes	Yes	Yes	Yes	Yes
**Triple combination:** **ERA + PDE5i + Treprostinil sc/iv or Epoprostenol iv**	Yes	Yes	Yes	Yes	Yes	Yes	Yes	Yes
**Quadruple combination:** **ERA + PDE5i + Treprostinil sc/iv or Epoprostenol iv + sotatercept**	Yes	Yes ^c^	No	No	No	No	No	No

^a^ Based on registry data; ^b^ based on estimation; ^c^ early access program only. Abbreviations: inh—inhalation, iv—intravenous, PAH—pulmonary arterial hypertension, po—per os, sc—subcutaneous.

**Table 2:** Comparison of age between adult patients receiving sildenafil and other PAH therapies * in 2024.

Gender	Sildenafil (Age M ± SD, *n*)	Other Treatment Regimens (Age M ± SD, *n*)
Male	63.6 ± 11 (28)	46.2 ± 18.2 (29)
Female	64 ± 15.9 (74)	54.2 ± 15.6 (85)
All	63.9 ± 14.8 (102)	52.2 ± 16.7 (114)

* All comparisons of patients’ age (male, female or all) between sildenafil and other treatment regimens were statistically significant (*p* < 0.05). Abbreviations: M—mean, SD—standard deviation, *n*—number of patients.

**Table 3:** Comparison of age between adult patients receiving sildenafil and other PAH therapies * in 2025.

Gender	Sildenafil (Age M ± SD, *n*)	Other Treatment Regimens (Age M ± SD, *n*)
Male	64 ± 10.8 (24)	43.5 ± 17.5 (28)
Female	65.5 ± 16.4 (61)	54.3 ± 15.2 (91)
All	65.1 ± 15.1 (85)	51.7 ± 16.4 (119)

* All comparisons of patients’ age (male, female or all) between sildenafil and other treatment regimens were statistically significant (*p* < 0.05). Abbreviations: M—mean, SD—standard deviation, *n*—number of patients.

**Table 4:** Patients’ age during diagnosis of PAH and duration of life during treatment.

Treatment Regimen	*n*	Age During Diagnosis (Years), M ± SD	Duration of Life Since Diagnosis, (Months), M ± SD	Change from Prior Therapy (%)
Monotherapy	105	67.15 ± 14.63	31.66 ± 25.01	-
Dual therapy	57	61.16 ± 16.33	43.09 ± 28.06	26.5 (from monotherapy)
Triple therapy	29	45.0 ± 21.57 *	59.88 ± 29.44	47.1 (from monotherapy) 28.0 (from dual therapy)

Abbreviations: M—mean, SD—standard deviation, *n*—number of patients; * *p* < 0.01 comparing age on triple therapy and on mono- or dual therapy.

**Table 5:** Statistical significance in patients’ duration of life during PAH treatment with different regimens.

Comparison	Raw *p*-Value	Bonferroni-Adjusted *p*-Value *
1 L vs. 2 L	0.0121	0.036
1 L vs. 3 L	0.000038	0.00011
2 L vs. 3 L	0.0154	0.046

Abbreviations: 1 L—monotherapy, 2 L—dual therapy, 3 L—triple therapy; * *p*-value < 0.05 indicates statistically significant results.

**Table 6:** Comparison of hazard ratios with and without age adjustment.

Comparison	HR (Unadjusted)	95% CI	HR (Adjusted for Age, Continuous)	95% CI
1 L vs. 2 L	1.471	1.063–2.036	1.536	1.089–2.165
3 L vs. 2 L	0.579	0.365–0.920	0.550	0.340–0.890
1 L vs. 3 L	2.540	1.644–3.925	2.792	1.698–4.589

Abbreviations: 1 L—monotherapy, 2 L—dual therapy, 3 L—triple therapy, HR—hazard ratio, CI—confidence interval.

## Data Availability

The original contributions presented in this study are included in the article. Further inquiries can be directed to the corresponding author.
